# Supplementary figures and images for: Enhanced thermal stability enables human mismatch-specific thymine–DNA glycosylase to catalyse futile DNA repair
Source: PLoS One. 2024 Oct 18;19(10):e0304818. doi: 10.1371/journal.pone.0304818 (PMC11488719; doi:10.1371/journal.pone.0304818)

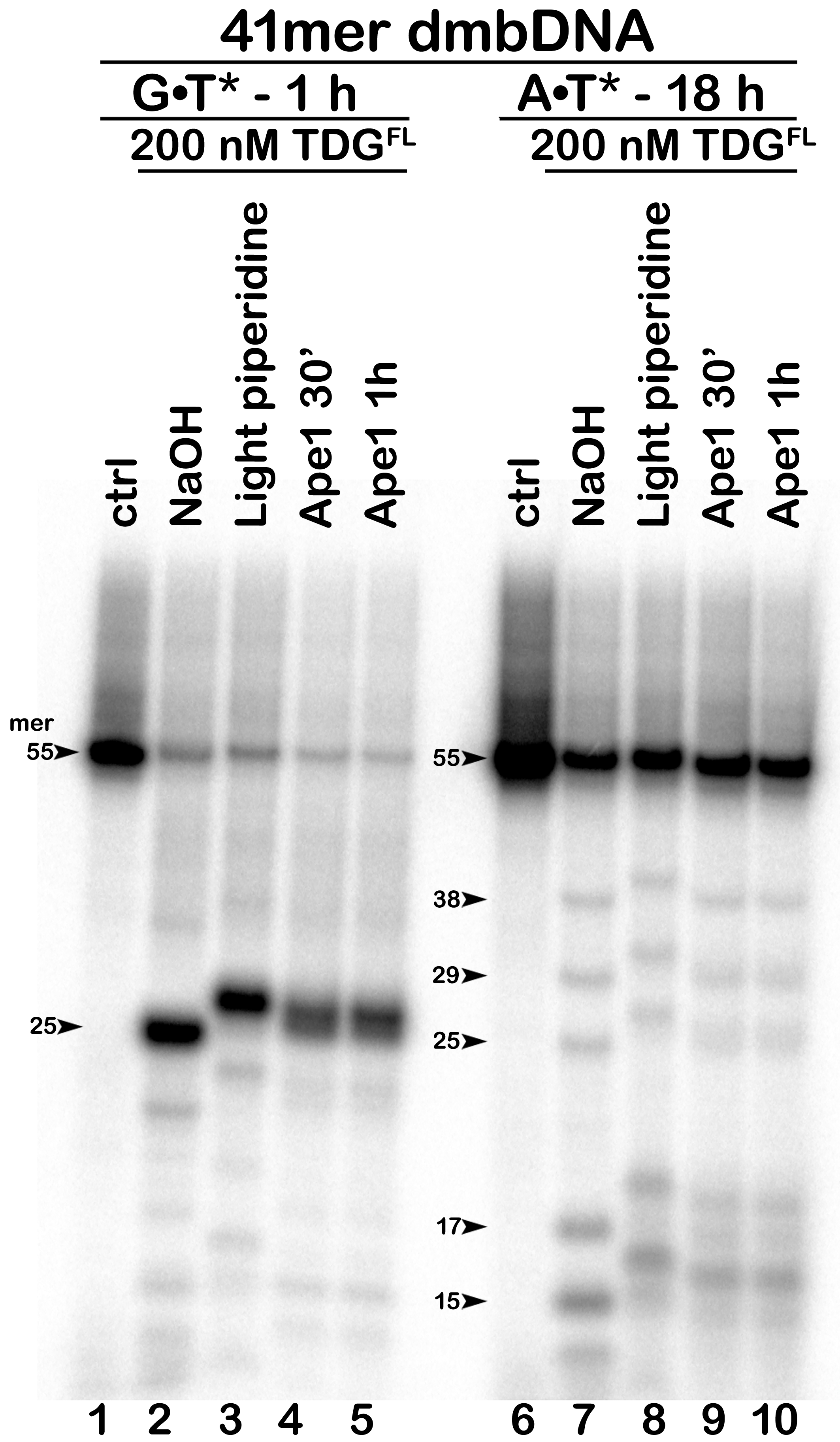

Supplement: S1 Fig — dmbDNA with the 32P-labelled bottom 55-mer strand containing T at position 26 opposite to G (lanes 1–5) or A (lanes 6–10) was incubated with TDGFL for 1 h (lanes 1–5) or 18 h (lanes 6–10) and post-treated with hot alkali (NaOH), light piperidine, or APE1 endonuclease. Lanes 1 and 6, no enzyme; lanes 2 and 7, TDGFL, then hot alkali; lanes 3 and 8, TDGFL, then light piperidine; lanes 4 and 9, TDGFL, then APE1 for 30 min; lanes 5 and 10, TDGFL, then APE1 for 1 h. Arrows mark the size of the DNA substrate and the cleavage fragments. For details, see Materials and Methods. (TIF) [file pone.0304818.s001.tif]

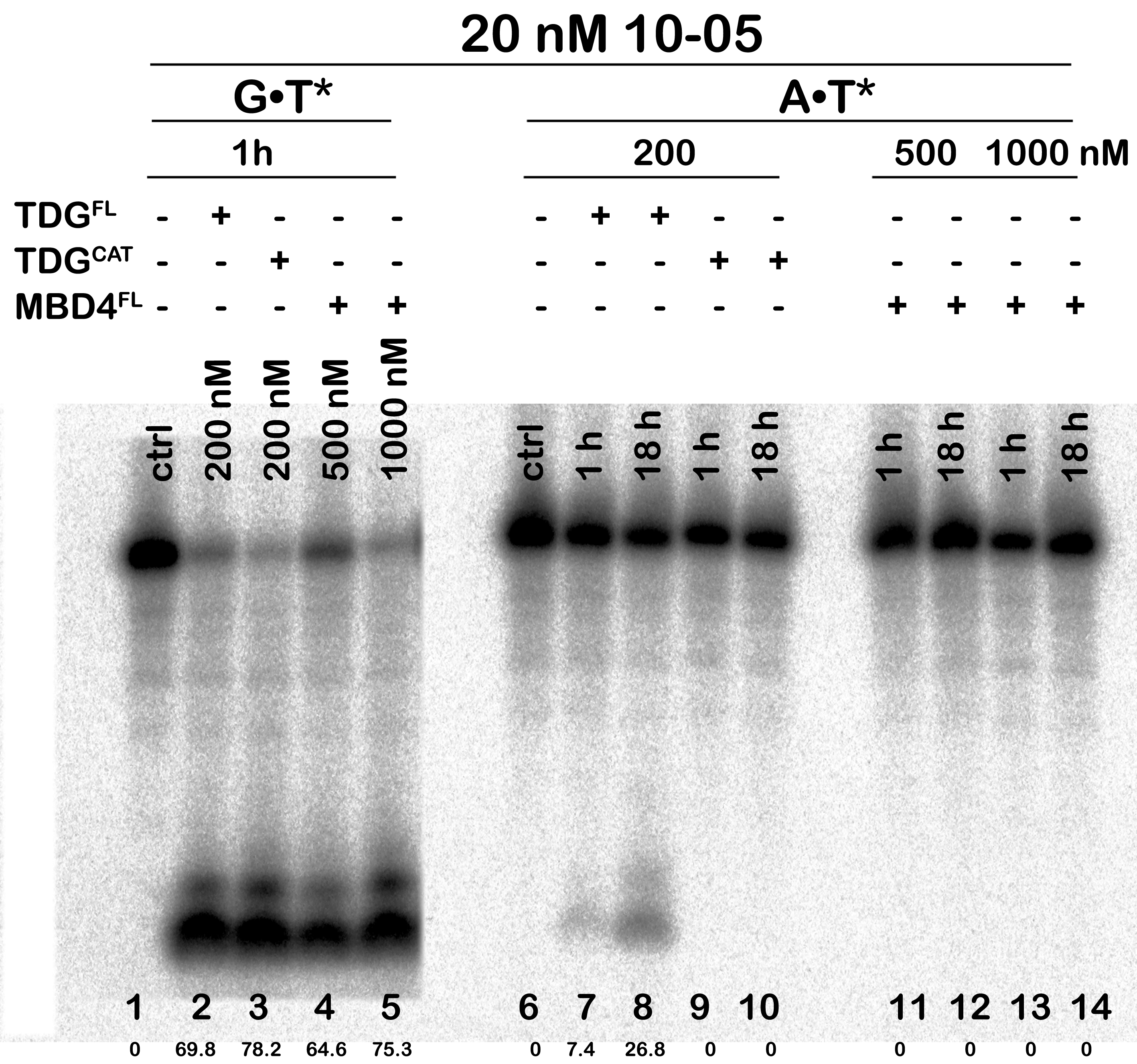

Supplement: S2 Fig — Denaturing PAGE analysis of the reaction products generated by TDGFL, TDGcat and MBD4 when acting upon 5′-32P-labelled 24-mer 10–05 G•T* and A•T* duplexes for 1 h and 18 h at 37°C. Percentage of cleavage products is indicated under the gel images. For details, see Materials and Methods. (TIF) [file pone.0304818.s002.tif]

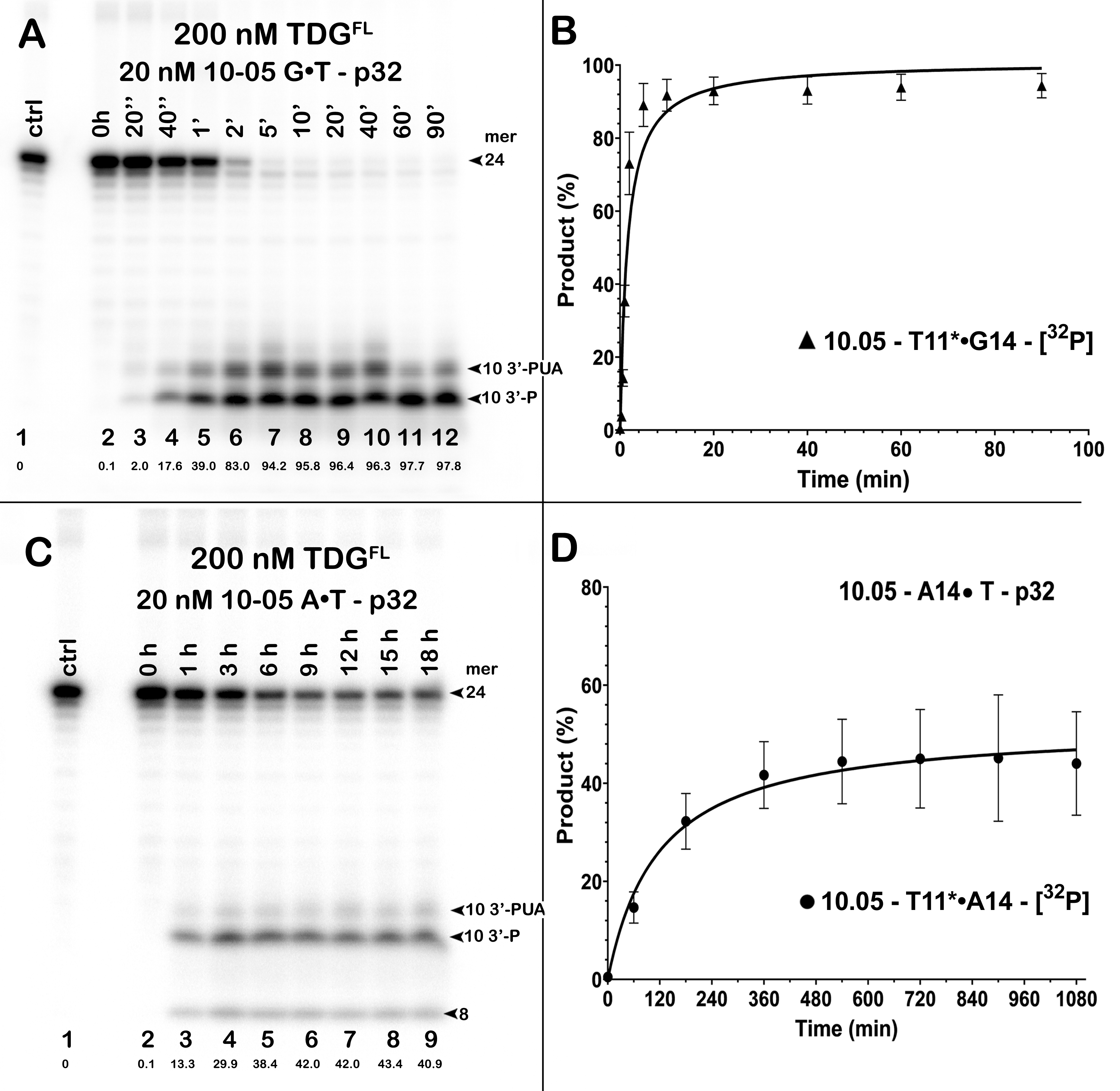

Supplement: S3 Fig — (A, C) Denaturing PAGE analysis of the reaction products. Time courses were performed at 37°C for 0–90 min (A) and 0–18 h (C) using 20 nM oligonucleotide duplexes where the T-containing strand is 5′-32P-labelled. Arrows mark the size of the DNA substrate and the cleavage fragments. Percentage of cleavage products is indicated under the gel images. (B, D) Plots of pre-steady-state single turnover kinetic of TDGFL-catalysed cleavage of 10–05 duplexes. Mean ± SD of three independent experiments is shown. For details, see Materials and Methods. (TIF) [file pone.0304818.s003.tif]

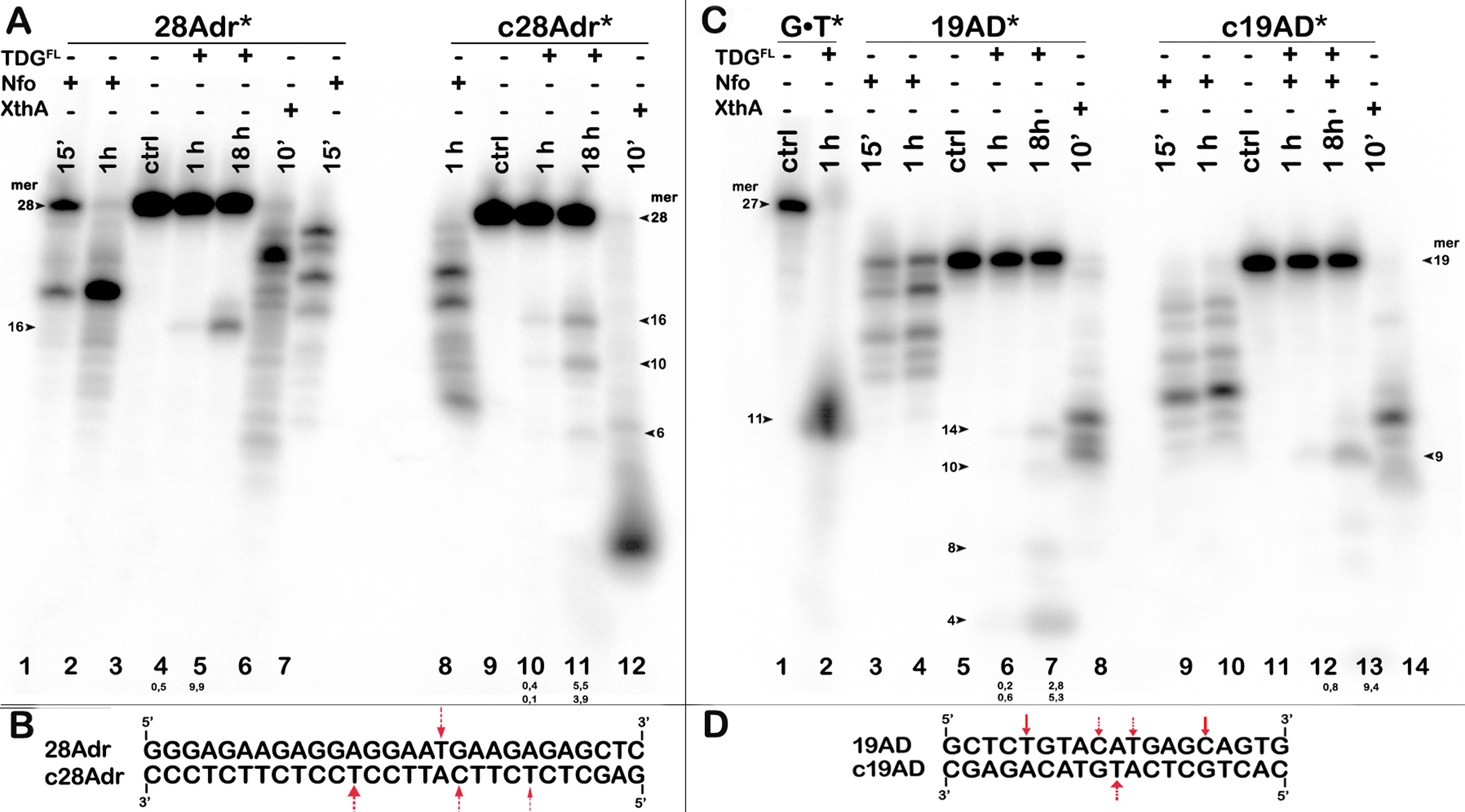

Supplement: S4 Fig — (A, C) Denaturing PAGE analysis of the reaction products. Arrows mark the size of the DNA substrate and the cleavage fragments. Percentage of cleavage products is indicated under the gel images. (A) Lanes 1–7, 28Adr strand is labelled; lanes 8–12, c28Adr strand is labelled. (C) Lanes 1–8, 19AD strand is labelled; lanes 9–14, c19AD strand is labelled (B, D) Schematic representation of 28Adr (B) and 19AD (D) sequences with red with arrows pointing to the pyrimidines excised by the enzyme. For details, see Materials and Methods. (TIF) [file pone.0304818.s004.tif]

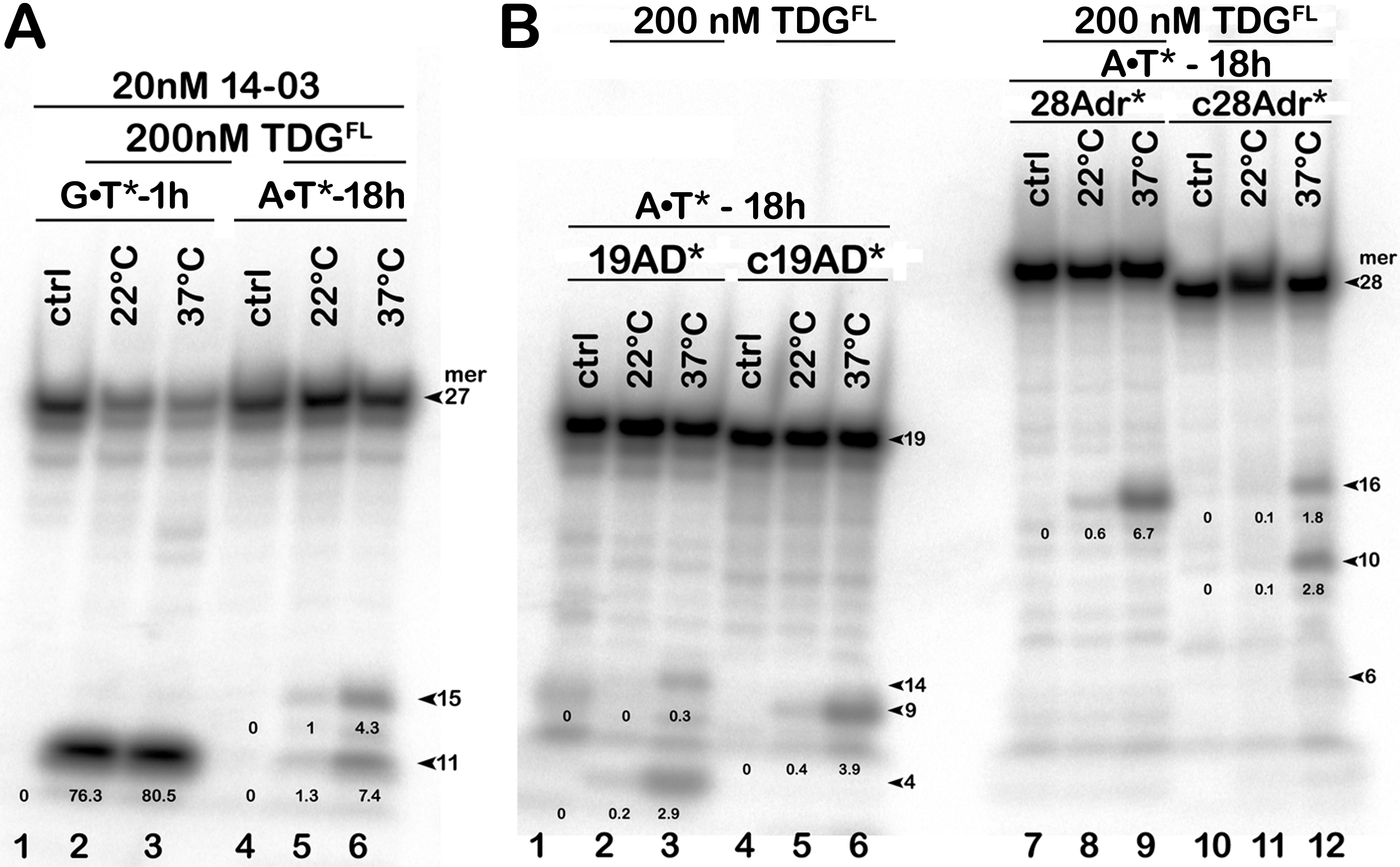

Supplement: S5 Fig — 27-mer 14–03 G•T* and A•T* duplexes, 28-mer 28Adr/c28Adr and 19-mer 19AD/c19AD A*•T and A•T* duplexes with either the top or the bottom DNA strand 32P-labelled were incubated with TDGFL for 1 h or 18 h at 22°C or 37°C. After the reaction, all samples including controls were treated by hot alkali. (A) Denaturing PAGE analysis of the products of 14–03 cleavage. Lanes 1–3, 14–03 G•T* duplex in which the top strand (T at position 12) is labelled: lane 1, no enzyme; lane 2, TDGFL for 1 h at 22°C; lane 3, TDGFL for 1 h at 37°C. Lanes 4–6, same as lanes 1–3 but regular 14–03 A•T* duplex. (B) Denaturing PAGE analysis of the cleavage products of 19AD (lanes 1–6) and 28Adr (lanes 7–12). Lanes 1–3, the 19AD strand is labelled: lane 1, no enzyme; lane 2, TDGFL for 18 h at 22°C; lane 3, TDGFL for 18 h at 37°C. Lanes 4–6, 7–9 and 10–12, same as lanes 1–3 but with c19AD, 28Adr and c28Adr strand labelled, respectively. Arrows mark the size of the DNA substrate and the cleavage fragments. Percentage of cleavage products is indicated under the gel images. For details, see Materials and Methods. (TIF) [file pone.0304818.s005.tif]

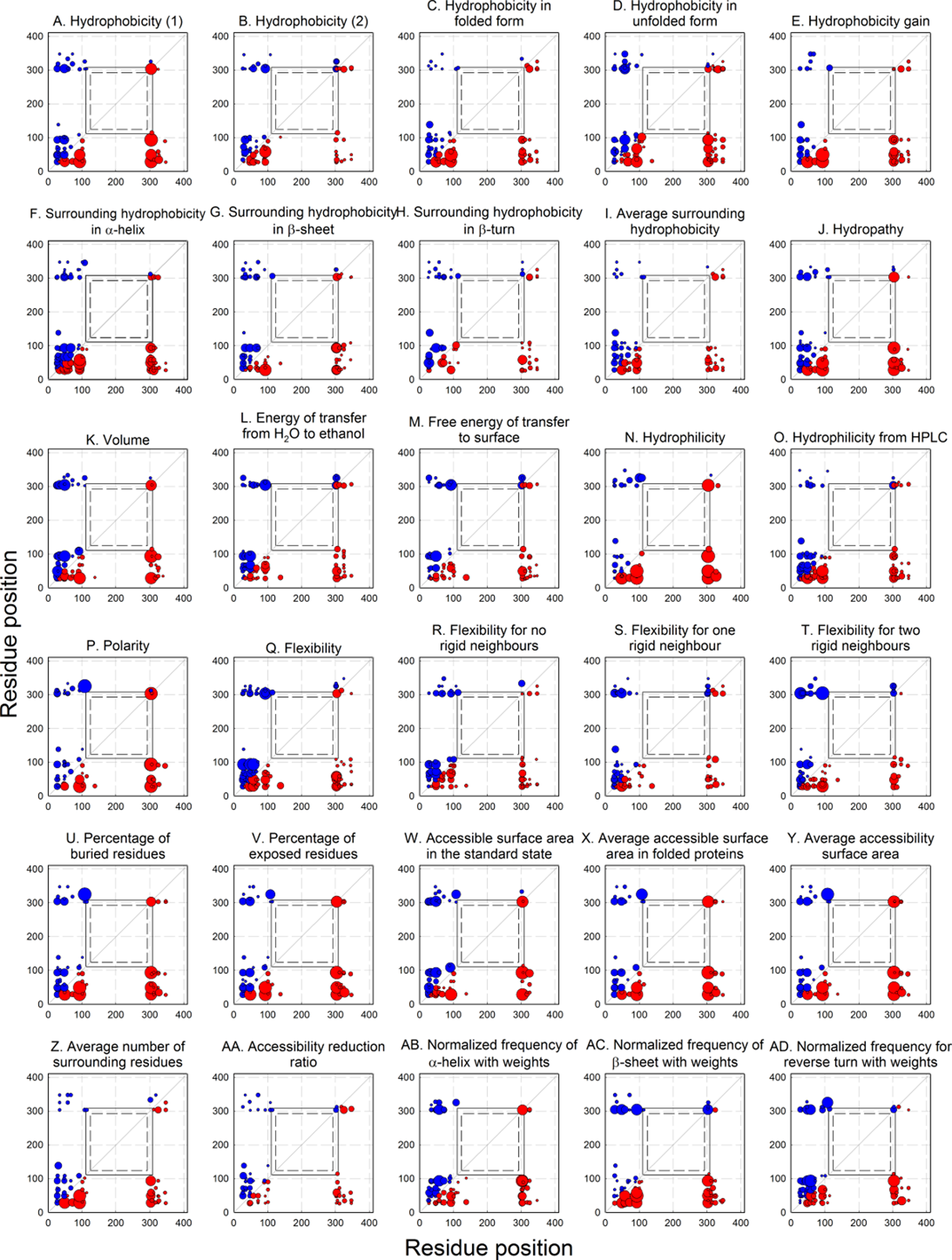

Supplement: S6 Fig — The amino acid properties analyzed for correlations were the overall hydrophobicity (A, B) [1, 2], hydrophobicity in folded (C) and unfolded form (D), hydrophobicity gain upon unfolding (E), surrounding hydrophobicity in α-helices (F), β-sheets (H) and β-turns (H) [3], average surrounding hydrophobicity (I) [4], hydropathy (J) [5], volume (K) [6], free energy of transfer from aqueous solution to ethanol (L) [7] and to surface (M) [8], hydrophilicity obtained from structural (N) [9] and HPLC data (O) [10], polarity (P) [3], overall flexibility (Q) [11], flexibility with none (R), one (S), or two rigid neighbours (T) [12], percentage of buried (U) and exposed residues (V) [13], accessible surface area in the standard state (W) and in the folded state (X) [14], average accessibility surface area (Y) [13], average number of surrounding residues (Z), accessibility reduction ratio (AA) [3], normalized frequency of α-helices (AB), β-sheets (AC) and reverse turns (AD) [15]. Red circles under the diagonal, positive correlation; blue circles above the diagonal, negative correlation; circle radii are proportional to the absolute value of correlation coefficient (rij) for the property for the given position pair. Only the pairs with p < 0.0001 (|rij| > 0.1947) are shown. The rectangles delimit the catalytic domain [16] (residues 111–308; solid lines) and the UDG-F2_TDG_MUG conserved domain as defined in the NCBI Conserved Domain Database [17] (residues 124–293; dashed lines). (TIF) [file pone.0304818.s006.tif]

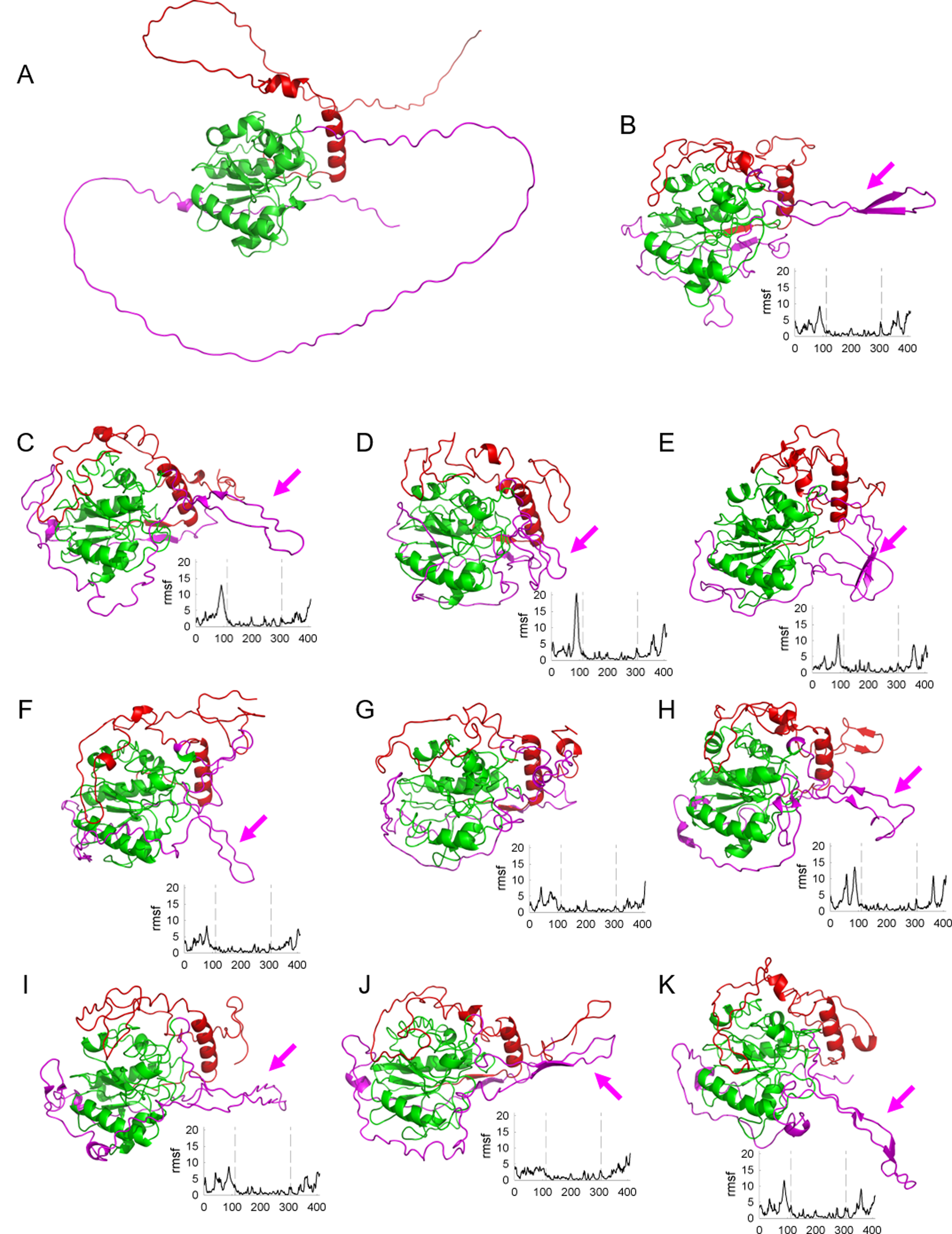

Supplement: S7 Fig — (A) and ten models (centroids of the most populated clusters) produced by independent CABS-flex runs (B–K). N-terminal tails are colored magenta, TDGcat, green, C-terminal tails, red. Arrows point to the hairpin-like structure in the N-terminal tail. Insets show r.m.s.f. profiles along the polypeptide chain, dashed lines delimit the catalytic domain. (TIF) [file pone.0304818.s007.tif]

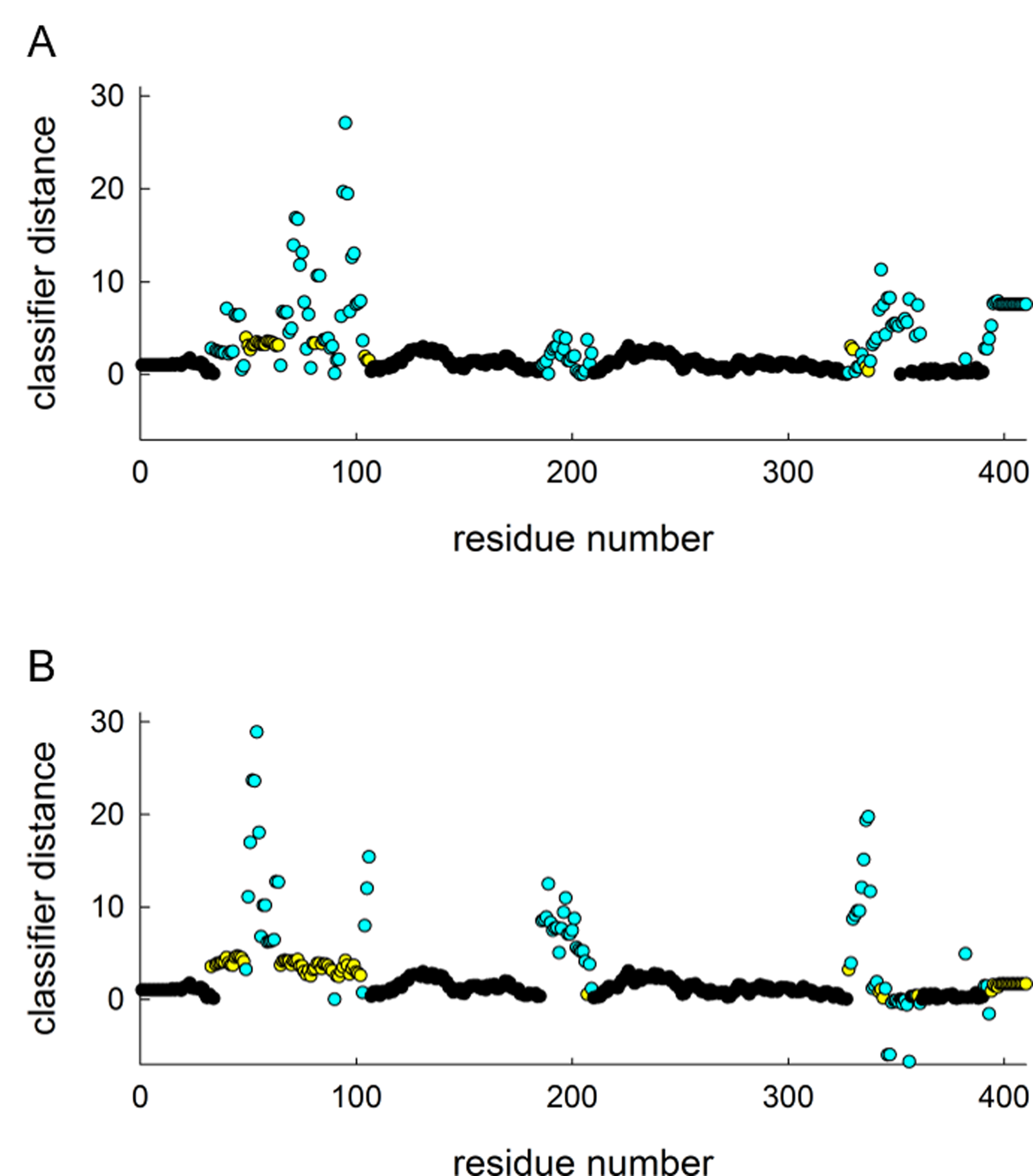

Supplement: S8 Fig — (A), classifier additionally trained to consider the standard molar enthalpy associated with phase separation, Δh°. (B), classifier additionally trained to consider the saturation concentration, csat. The color code is: cyan, residues intrinsically disordered and prone to undergo phase separation; yellow, intrinsically disordered but do not undergo phase separation; black, may or may not be intrinsically disordered but can fold to a stable conformation. (TIF) [file pone.0304818.s008.tif]

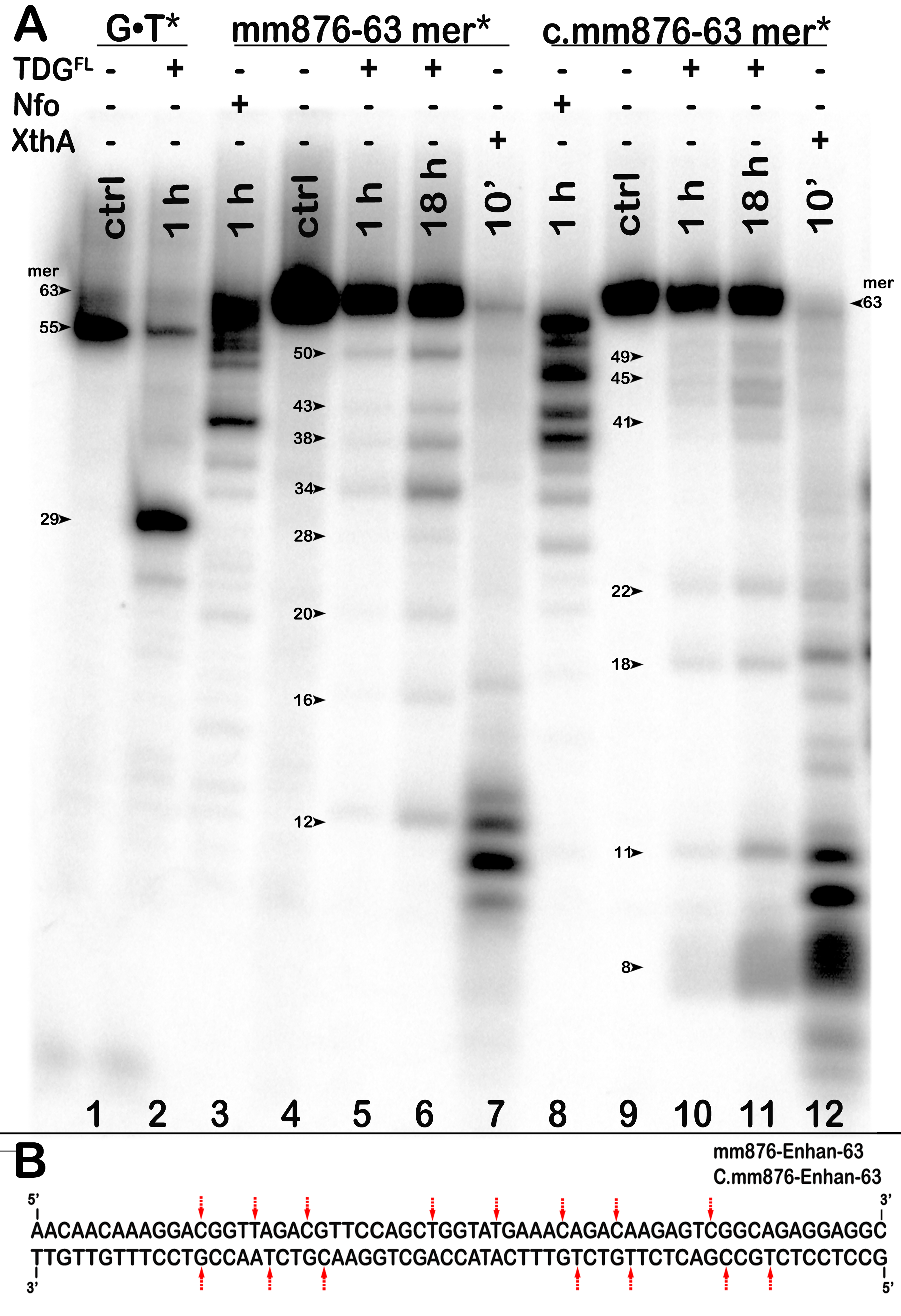

Supplement: S9 Fig — (A) Denaturing PAGE analysis; 63-mer duplexes in which either the top or the bottom DNA strand is 5′-32P-labelled were incubated with TDGFL for 1 h or 18 h at 37°C. Lanes 1–2, 63-mer T*•G duplex (strands 63 and c63, Table 1); lanes 3–7, mm876 duplex in which the mm876 strand is labelled; lanes 8–12, mm876 duplex in which the c.mm876 strand is labelled. 3′→5′ exonuclease degradation of the mm876 duplexes by Nfo and Xth and TDG cleavage of a 63-mer T*•G duplex were used to generate size markers. Arrows mark the size of the DNA substrate and the cleavage fragments. For details, see Materials and Methods. (B) Schematic representation of the mm876 sequence with red arrows pointing to the pyrimidines excised by the enzyme. (TIF) [file pone.0304818.s009.tif]

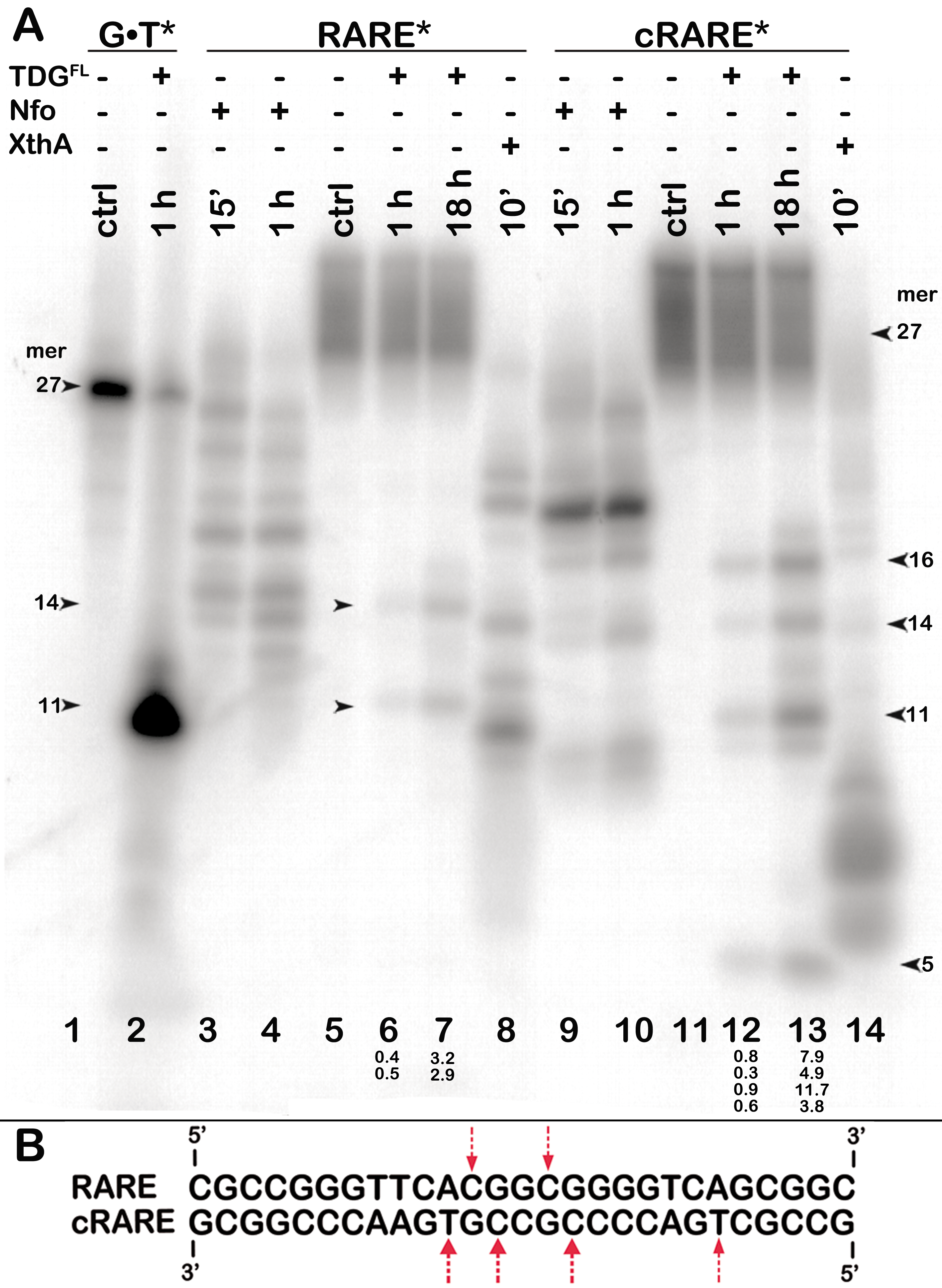

Supplement: S10 Fig — (A) Denaturing PAGE analysis; 27-mer duplexes in which either the top or the bottom DNA strand is 5′-32P-labelled were incubated with TDGFL for 1 h or 18 h at 37°C. Lanes 1–2, 27-mer T*•G duplex; lanes 3–8, RARE duplex in which the RARE strand is labelled; lanes 9–14, RARE duplex in which the c.RARE strand is labelled. 3′→5′ exonuclease degradation of the RARE duplexes by Nfo and Xth and TDG cleavage of a 27-mer T*•G duplex were used to generate size markers. Arrows mark the size of the DNA substrate and the cleavage fragments. Percentage of cleavage products is indicated under the gel images. For details, see Materials and Methods. (B) Schematic representation of the RARE sequence with red with arrows pointing to the pyrimidines excised by the enzyme. (TIF) [file pone.0304818.s010.tif]

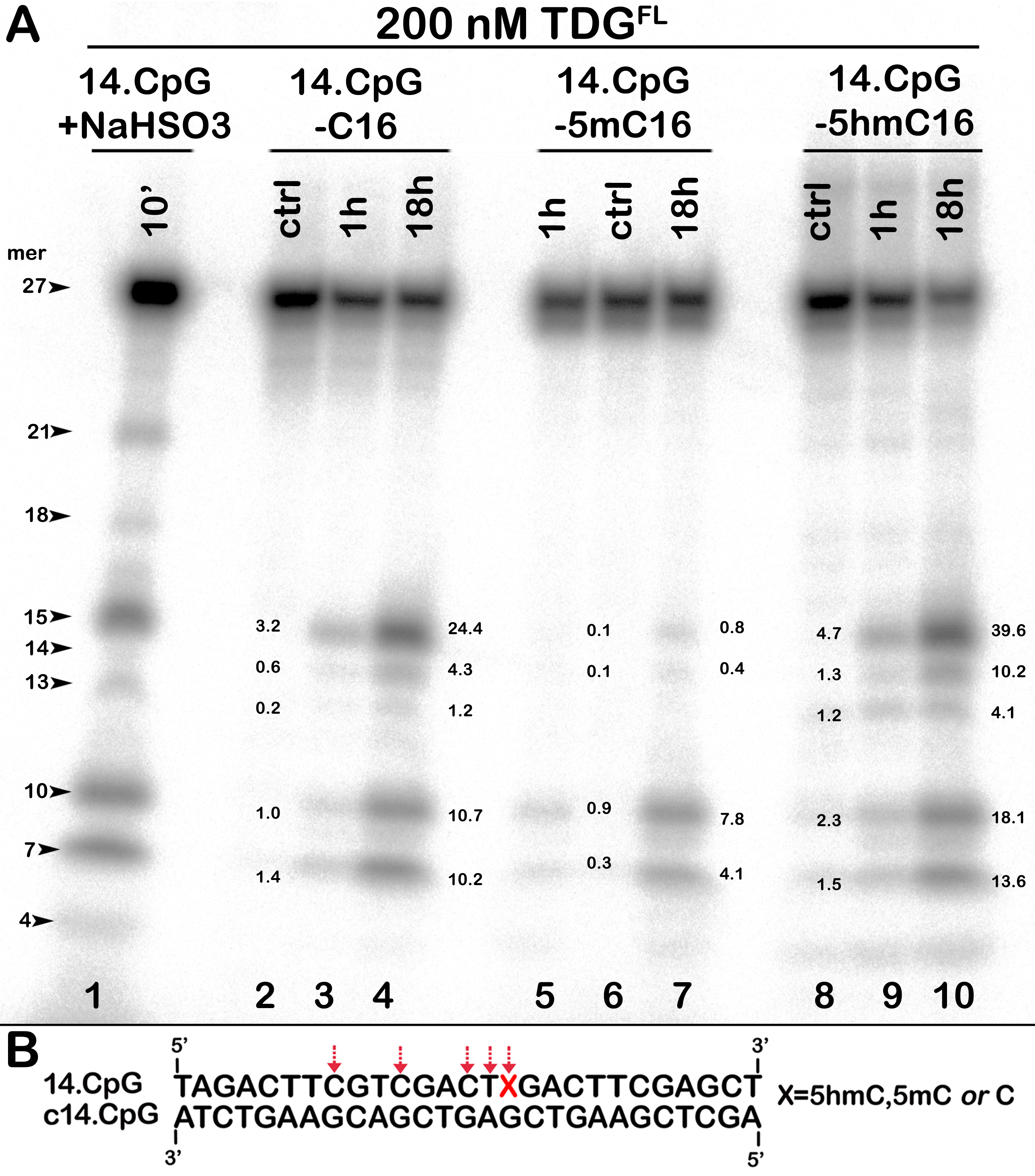

Supplement: S11 Fig — (A) Denaturing PAGE analysis; 5′-32P-labelled 14.CpG C*•G, 5hmC*•G and 5mC*•G duplexes were incubated with TDGFL for 1 h or 18 h at 37°C. Lane 1, bisulfite-treated single-stranded 14.CpG oligonucleotide was incubated with UNG to generate size markers corresponding to cytosine positions; lanes 2–4, 14.CpG C*•G duplex; lanes 5–7, 14.CpG 5mC*•G duplex; lanes 8–10, 14.CpG 5hmC*•G duplex. Arrows indicate the size of DNA substrate and cleavage products. The numbers next to the bands correspond to the percentage of cleavage products For details, see Materials and Methods. (B) Schematic representation of the 14.CpG sequence with red with arrows pointing to the pyrimidines excised by the enzyme. (TIF) [file pone.0304818.s011.tif]
