# Supplementary material for: Enhanced thermal stability enables human mismatch-specific thymine–DNA glycosylase to catalyse futile DNA repair
Source: PLoS One. 2024 Oct 18;19(10):e0304818. doi: 10.1371/journal.pone.0304818 (PMC11488719; doi:10.1371/journal.pone.0304818)
Supplement: S1 File — (DOCX) [file pone.0304818.s013.docx]

**Supporting References**

1. Eisenberg D, Schwarz E, Komaromy M, Wall R. Analysis of membrane and surface protein sequences with the hydrophobic moment plot. J Mol Biol. 1984; 179:125-142. <https://doi.org/10.1016/0022-2836(84)90309-7> PMID: 6502707
2. Jones DD. Amino acid properties and side-chain orientation in proteins: A cross correlation approach. J Theor Biol. 1975; 50:167-183. <https://doi.org/10.1016/0022-5193(75)90031-4> PMID: 1127956
3. Ponnuswamy PK, Prabhakaran M, Manavalan P. Hydrophobic packing and spatial arrangement of amino acid residues in globular proteins. Biochim Biophys Acta. 1980; 623:301-316. <https://doi.org/10.1016/0005-2795(80)90258-5> PMID: 7397216
4. Manavalan P, Ponnuswamy PK. Hydrophobic character of amino acid residues in globular proteins. Nature. 1978; 275:673-674. <https://doi.org/10.1038/275673a0> PMID: 1978-10-19
5. Kyte J, Doolittle RF. A simple method for displaying the hydropathic character of a protein. J Mol Biol. 1982; 157:105-132. <https://doi.org/10.1016/0022-2836(82)90515-0> PMID: 7108955
6. Chothia C. Principles that determine the structure of proteins. Annu Rev Biochem. 1984; 53:537-572. <https://doi.org/10.1146/annurev.bi.53.070184.002541> PMID: 6383199
7. Nozaki Y, Tanford C. The solubility of amino acids and two glycine peptides in aqueous ethanol and dioxane solutions: Establishment of a hydrophobicity scale. J Biol Chem. 1971; 246:2211-2217. <https://doi.org/10.1016/S0021-9258(19)77210-X> PMID: 5555568
8. Bull HB, Breese K. Surface tension of amino acid solutions: A hydrophobicity scale of the amino acid residues. Arch Biochem Biophys. 1974; 161:665-670. <https://doi.org/10.1016/0003-9861(74)90352-X> PMID: 4839053
9. Hopp TP, Woods KR. Prediction of protein antigenic determinants from amino acid sequences. Proc Natl Acad Sci USA. 1981; 78:3824-3828. <https://doi.org/10.1073/pnas.78.6.3824> PMID: 6167991
10. Parker JMR, Guo D, Hodges RS. New hydrophilicity scale derived from high-performance liquid chromatography peptide retention data: Correlation of predicted surface residues with antigenicity and X-ray-derived accessible sites. Biochemistry. 1986; 25:5425-5432. <https://doi.org/10.1021/bi00367a013> PMID: 2430611
11. Bhaskaran R, Ponnuswamy PK. Positional flexibilities of amino acid residues in globular proteins. Int J Pept Protein Res. 1988; 32:241-255. <https://doi.org/10.1111/j.1399-3011.1988.tb01258.x>
12. Karplus PA, Schulz GE. Prediction of chain flexibility in proteins: A tool for the selection of peptide antigens. Naturwissenschaften. 1985; 72:212-213. <https://doi.org/10.1007/BF01195768>
13. Janin J, Wodak S, Levitt M, Maigret B. Conformation of amino acid side-chains in proteins. J Mol Biol. 1978; 125:357-386. <https://doi.org/10.1016/0022-2836(78)90408-4> PMID: 731698
14. Rose GD, Geselowitz AR, Lesser GJ, Lee RH, Zehfus MH. Hydrophobicity of amino acid residues in globular proteins. Science. 1985; 229:834-838. <https://doi.org/10.1126/science.4023714> PMID: 4023714
15. Levitt M. Conformational preferences of amino acids in globular proteins. Biochemistry. 1978; 17:4277-4285. <https://doi.org/10.1021/bi00613a026> PMID: 708713
16. Steinacher R, Schär P. Functionality of human thymine DNA glycosylase requires SUMO-regulated changes in protein conformation. Curr Biol. 2005; 15:616-623. <https://doi.org/10.1016/j.cub.2005.02.054> PMID: 15823533
17. Marchler-Bauer A, Derbyshire MK, Gonzales NR, Lu S, Chitsaz F, Geer LY, et al. CDD: NCBI’s conserved domain database. Nucleic Acids Res. 2015; 43:D222-D226. <https://doi.org/10.1093/nar/gku1221> PMID: 25414356
18. Ibrahim AY, Khaodeuanepheng NP, Amarasekara DL, Correia JJ, Lewis KA, Fitzkee NC, et al. Intrinsically disordered regions that drive phase separation form a robustly distinct protein class. J Biol Chem. 2023; 299:102801. <https://doi.org/10.1016/j.jbc.2022.102801> PMID: 36528065
