# Supplementary material for: Enhanced thermal stability enables human mismatch-specific thymine–DNA glycosylase to catalyse futile DNA repair
Source: PLoS One. 2024 Oct 18;19(10):e0304818. doi: 10.1371/journal.pone.0304818 (PMC11488719; doi:10.1371/journal.pone.0304818)
Supplement: Attachment — Submitted filename: Manapkyzy PLOS One Review 2024-06-10.docx [file pone.0304818.s014.docx]

Manapkyzy and colleagues have prepared a manuscript entitled Enhanced thermal stability enables human mismatch-specific Thymine-DNA glycosylase to catalyse futile DNA repair. This manuscript suggests that full-length human TDG shows improved enzyme stability as demonstrated by increased activity especially in regards to length of enzyme activity. They further propose that the full-length enzyme is able to excise 5-hydroxymethylcytosine (5hmC), but not 5- methylcytosine residues, and is more active than previously described on T opposite A compared to its preferred substrate T opposite G.

**Major comments:**

The most contentious argument the authors make is that 5hmC is excised by human TDG and to a lesser extent T:A, and C:G. This has not been previously demonstrated by any group regardless of full-length or partial-length TDG usage. Because of their disagreement with the literature, I feel that the bar to demonstrate this argument is not sufficiently met with the current data provided. If the authors intend to claim this, I think they should include the catalytic rate constants of all proposed substrates, including 5hmC, as they do in Table 2, to determine if other claimed activities are real or not. Furthermore, other groups have used full-length TDG and not seen these activities under similar conditions albeit no incubation for 18 h (1).

This manuscript proposes excision of T opposite A and C excision opposite G. Figure 1 first provides evidence of this, but it is only seen in lanes treated with piperidine but not the bands treated with NaOH. This seems to suggest an artifact and is not sufficiently convincing evidence to me. The authors could consider using APE1 as an additional control lane as it would not require chemical backbone cleavage or reduce radioisotope band intensity.

The naming conventions of their substrates is arbitrary to readers and this reviewer, but only adds confusion. This makes it very challenging to easily interpret their findings and if it agrees with their proposed arguments. I feel this is a major comment as the manuscript is difficult to interpret.

TDG full-length being more active/stable than just the catalytic domain alone has already been described by the Drohat group as well. This is consistent with literature and the Drohat group demonstrated that at least a 29-amino acid portion of the intrinsically disordered N-terminal domain was important for improving activity of the expressed enzyme and reconstituted full-length activity (1, 2). This argument already exists in the literature, and I feel if the authors work also supports this they should describe it in the context of existing literature.

**Minor comments:**

Figure 2 is non-contributory and interpretation of very minor bands. I do not feel this adds anything to their manuscript.

What is the purpose of using the dumbbell shaped substrate rather than a simple duplex? Why do the authors mix and match fluorescent oligonucleotide data with traditional P32 isotope labeling experiments? Perhaps it may not need to go into the manuscript, but this reviewer is unclear as to some of the experimental design/decisions.

**References**

1.	Baljinnyam, T., Sowers, M. L., Hsu, C. W., Conrad, J. W., Herring, J. L., Hackfeld, L. C., and Sowers, L. C. (2022) Chemical and enzymatic modifications of 5-methylcytosine at the intersection of DNA damage, repair, and epigenetic reprogramming. *PLoS One*. **17**, e0273509

2.	Coey, C. T., Malik, S. S., Pidugu, L. S., Varney, K. M., Pozharski, E., and Drohat, A. C. (2016) Structural basis of damage recognition by thymine DNA glycosylase: Key roles for N-terminal residues. *Nucleic Acids Res.* **44**, 10248–10258
