# Supplementary material for: Enhanced thermal stability enables human mismatch-specific thymine–DNA glycosylase to catalyse futile DNA repair
Source: PLoS One. 2024 Oct 18;19(10):e0304818. doi: 10.1371/journal.pone.0304818 (PMC11488719; doi:10.1371/journal.pone.0304818)
Supplement: Attachment — Submitted filename: PONE-D-24-19416 reviewer comments.pdf [file pone.0304818.s015.pdf]

The introduction states that TDG is known for its wide DNA substrate specificity, excising mismatched DNA substrates, while MBD4 has a narrow DNA substrate specificity. Do the authors base this statement solely on the number of substrates excised by these enzymes? The enzyme activities in prior works have not been compared on a common footing, as those experiments used different initial conditions and setups. It might be better to present this as a conjecture.

Other challenges related to TDG's conformational stability led to a kinetics experiment of BER at 22°C. It is known that non-specific DNA in the reaction buffer stabilizes TDG conformation against heat-induced unfolding at 37°C. This has led to the conjecture that DNA binding prevents protein aggregation, enhances thermal stability, and modulates specificity.

Nonspecific DNA-stabilized TDG catalyzes the aberrant removal of T paired with A, as well as C, 5mC, and 5hmC residues paired with G in non-damaged DNA duplexes. This has been investigated to determine the kinetics of excision. However, it is not entirely fair to presume that specificity can identically discriminate the various derivatives of cytosine at both 22°C and 37°C.
